# Estimating common dolphin bycatch in the pole-and-line tuna fishery in the Azores

**DOI:** 10.7717/peerj.4285

**Published:** 2018-02-12

**Authors:** Maria João Cruz, Miguel Machete, Gui Menezes, Emer Rogan, Mónica A. Silva

**Affiliations:** 1 Departamento de Oceanografia e Pescas, Universidade dos Açores, Horta, Açores, Portugal; 2 MARE—Marine and Environmental Sciences Centre and IMAR—Instituto do Mar, Universidade dos Açores, Horta, Açores, Portugal; 3 School of Biological, Earth and Environmental Sciences, University College Cork, Enterprise Centre, Distillery Fields, North Mall, Cork, Ireland; 4 Department of Biology, Woods Hole Oceanographic Institution, Woods Hole Oceanographic Institution, Woods Hole, MA, USA

**Keywords:** Bycatch, Cetacean conservation, Fishery interaction, Statistical modelling, *Delphinus delphis*, Azores

## Abstract

Small-scale artisanal fisheries can have a significant negative impact in cetacean populations. Cetacean bycatch has been documented in the pole-and-line tuna fishery in the Azores with common dolphins being the species more frequently taken. Based on data collected by observers on ∼50% of vessels operating from 1998 to 2012, we investigate the influence of various environmental and fisheries-related factors in common dolphin bycatch and calculate fleet-wide estimates of total bycatch using design-based and model-based methods. Over the 15-year study dolphin bycatch occurred in less than 0.4% of the observed fishing events. Generalized additive modelling results suggest a significant relationship between common dolphin bycatch and duration of fishing events, sea surface temperature and location. Total bycatch calculated from the traditional stratified ratio estimation approach was 196 (95% CI: 186–205), while the negative binomial GAM estimated 262 (95% CI: 249–274) dolphins. Bycatch estimates of common dolphin were similar using statistical approaches suggesting that either of these methods may be used in future bycatch assessments for this fishery. Our work shows that rates of common dolphin bycatch in the pole-and-line tuna fishery in the Azores are low, despite considerable variations between years. Dolphins caught were released alive although the fate of these individuals is unknown. Continued monitoring will provide a better understanding of dolphin bycatch and more accurate estimates essential in the development of potential mitigation measures.

## Introduction

The incidental catch of non-target marine mammals in fisheries, termed bycatch, is a worldwide problem, with several hundred thousand animals killed per year across the globe (Lewison et al., 2004; Read, Drinker & Northridge, 2006; Reeves, McClellan & Werner, 2013; Briscoe et al., 2014). Cetaceans are particularly vulnerable to the effects of bycatch mortality due to the low rates of potential population growth (Read, Drinker & Northridge, 2006; Read, 2008; Wade, Reeves & Mesnick, 2012; Geijer & Read, 2013).

Bycatch of marine mammals occurs in nearly all gears, with gillnets being responsible for the highest mortality, followed by trawls and longlines, particularly in the eastern Pacific Ocean and Mediterranean Sea (Read, Drinker & Northridge, 2006; Soykan et al., 2008; Lewison et al., 2014). Most of the bycatch occurs in industrial fisheries although bycatch in small-scale artisanal fisheries can be substantial (Soykan et al., 2008; Reeves, McClellan & Werner, 2013; Lewison et al., 2014). About 2,500 small dolphins are captured annually off the coast of Peru by the artisanal drift gillnet and longline fisheries (Mangel et al., 2010). In Ecuador, 2,500–5,000 small cetaceans may be killed every year in artisanal longline and surface gillnet fisheries (Félix & Samaniego, 1994) and entanglement of humpback whales in artisanal gillnets may pose a serious threat for the survival of the population (Alava, Barragán & Denkinger, 2012; Alava et al., 2017). Incidental catches of dolphins in small-scale fisheries in East Malaysia exceed the maximum 2% annual anthropogenic removal tolerated by the population according to the potential biological removal (PBR), and may therefore be unsustainable (Jaaman, Lah-Anyi & Pierce, 2009).

The tuna fishery is one of the most important fisheries in the archipelago of the Azores. In 2012, 7,000t of tuna were landed by this fishery accounting for 60% of total landings (Machete, Morato & Santos, 2012) and for 48% of the economic revenue of the fishing sector (INE, 2013). The tuna fleet represents around 4% of the Azorean fishing fleet and employs about 17% of fishermen (Carvalho, Edwards-Jones & Isidro, 2011). This fishery uses pole-and-line, usually with water spray and live bait, and target species are the bigeye tuna (*Thunus obesus*), skipjack (*Katsuwonus pelamis*), albacore (*Thunus alalunga*), yellowfin (*Thunus albacares*) and bluefin tuna (*Thunus thynnus*) (Morato et al., 2008; Pham et al., 2013). Up until the late 1990s, dolphins were occasionally hunted by this fishery and their meat was ground up and used as chum for catching live bait such as mackerel (*Trachurus picturatus*) or sardine (*Sardina pilchardus*) used in tuna fishery. The Azorean Fisheries Observer Programme (POPA) was created in 1998 to monitor the fishery and to ensure the validity of the ‘dolphin safe’ certificate for tuna caught in the Azores. The program covered about half of the tuna fishing vessels and, in general, >50% of the tuna catches, which is the minimum required to certify the fishery (Morato et al., 2008).

Silva et al. (2002, 2011) used data collected by POPA programme to document dolphin bycatch in the Azorean tuna fishery between 1998 and 2006. Bycatch was observed in less than 1% of fishing events with common dolphins (*Delphinus delphis*) being the species more frequently taken. There are no records of cetacean mortality associated with this fishery, because all the animals were released alive by cutting the fishing line, although their post-release fate was uncertain. These authors used simple ratio estimators that are commonly used to extrapolate bycatch estimates as the product of observed bycatch rate (i.e. number of catches per observed fishing set or trip) and total effort in a fishery (i.e. number of sets or trips). They estimated that about 110 dolphins were captured by the tuna fishing fleet in the nine-year period (1998–2006).

The rarity of observed dolphin bycatch makes it difficult to conduct a rigorous statistical evaluation. In recent years, several approaches have been developed to deal with the analytical challenges posed by rare-event data to ensure that total bycatch estimated from these few observations is robust. The use of stratification schemes in ratio estimate methods may improve the precision of bycatch estimates, since, in addition to fishing effort, they can take into account other factors (e.g. gear characteristics, time and location of fishing, environmental conditions) believed to influence dolphin bycatch rates. However, ratio estimates may be biased by the uneven distribution of observer coverage across space and over time (Murray, 2007; Lawrence et al., 2009).

Model-based approaches that incorporate variables predicting dolphin bycatch can reduce the uncertainty of bycatch estimates by better accounting for biases in observer data (Lawrence et al., 2009). Although describing the relationship between dolphin bycatch and covariates may be challenging, especially when number of bycatch observations is low (Barry & Welsh, 2002), predictive models are increasingly used in bycatch assessments (Rossman, 2010; Thompson, Abraham & Berkenbusch, 2013; Brown, Reid & Rogan, 2014).

To assess dolphin bycatch in the pole-and-line fishery in the Azores we analysed data collected by POPA within the exclusive economic zone (EEZ) of the archipelago of the Azores between 1998 and 2012. We focused on common dolphins as this was the species involved in most bycatch reports. The objectives of this study were to (i) update information from Silva et al. (2011) on the frequency of common dolphin bycatch using a longer time series, (ii) understand which environmental and fisheries-related factors drive common dolphin bycatch, (iii) compare ratio and model-based estimators of bycatch, and (iv) provide improved estimates of common dolphin bycatch in the pole-and-line tuna fishery in the Azores. This knowledge is critical to evaluate the need of management measures and for the development of mitigation measures.

## Methods

### Study area

The Archipelago of the Azores (Portugal) is a group of nine volcanic islands situated in the North Atlantic, between 37° and 41°N and 25° and 31°W, crossing the Mid-Atlantic Ridge (Santos et al., 1995). The archipelago is divided into three groups: the Eastern group (Santa Maria and São Miguel), the Central group (Terceira, São Jorge, Graciosa, Pico and Faial) and the Western group (Flores and Corvo). Due to the scattered configuration of the islands, the archipelago has an extensive EEZ of almost one million square kilometres. The islands and their contiguous shelf (<500 m depth) represent about 0.4% of the Azores EEZ (Santos et al., 1995). Therefore, potential fishing grounds are scarce and limited to the narrow area of shallow water around the islands and to nearby banks and seamounts. The tuna fishery generally occurs around the island slopes and at seamounts but distribution of fishing effort varies considerable between years (Silva et al., 2002; Morato et al., 2008).

### Fishery observer data

Data were collected by POPA observers placed onboard commercial tuna vessels (>20 m length) from 1998 to 2012. A complete description of the tuna fishery and of POPA program and data collection can be found elsewhere (Silva et al., 2002; Machete & Santos, 2007; Cruz et al., 2016). The fishery occurs between April and November and each boat spends an average of eight days at sea before landing (Silva et al., 2002; Morato et al., 2008). Depending on tuna abundance and size of the schools encountered, there may be several fishing events during a fishing day. Fishing events are defined as when fishers are effectively capturing tuna. All the tuna fishing vessels operating in the Azores use the pole-and-line fishing technique. This fishery uses different fishing gear configurations, depending on the behaviour and morphology of the target species and on the distance or depth of the school (Feio & Dias, 2000). These gears differ in the length and type of nylon leader and pole used but all have a hook in the extremity of the line or pole and potentially represent a risk to cetaceans.

A single observer is assigned to each vessel for a 30-day period, after which observers rotate between fishing vessels. Observers are required to monitor all fishing events. For each fishing event, observers record the following information: location, beginning and end time of the event, number and type of gear used, number, size and weight of tuna captured, interaction and bycatch of cetaceans, seabirds and turtles. Tunas are measured at the end of each fishing event. Dolphin bycatch occurs when animals mix with the tuna school to feed on the live bait and accidentally bite or get stuck by the hooks in the fishing lines.

Fishing effort was calculated as the number of fishing events per trip (lasting eight days on average) and per vessel.

### Environmental data

The environmental variables selected for this study were sea surface temperature (SST), depth, distance to coast, and a proxy for prey abundance. These variables were selected because they are known to affect the abundance and distribution of many marine taxa, including dolphins, tunas and their potential prey (Brill & Lutcavage, 2001; Erzini, 2005; Hastie et al., 2005). Chlorophyll was not used since there were no data available prior to 2002.

Sea surface temperature at each fishing event was obtained from in situ measurements by the vessel echosounder. When SST values were not available for a fishing event, we used the average water temperature for the month. The predictor variables depth (m) and distance to coast (km) of the fishing event were calculated in ArcGIS. Depth was obtained as bathymetric data composite using multiple sources: GEBCO_08 (Lourenço et al., 1998), grid (MOMARGIS v2, DOP/UAz), multi-beam surveys (GMRT grids), point and contour data digitized from nautical charts in the vicinity of the islands. Original grid resolution varied between 50 m and 0.5′. Distance to coast was calculated in ArcGIS as the shortest distance between the coordinates point and the closest point on the coastline.

To investigate the effect of prey availability on the probability of common dolphin bycatch, we used landings statistics of locally abundant species that likely serve as dolphin prey, since there are no fisheries-independent abundance estimates of small pelagic species. Landings of bogue (Boops boops), sardine, chub mackerel (Scomber colias) and horse mackerel (Lotaçor, 2014) were pooled together for each month of the study period and used as a proxy for prey abundance in the region.

### Analysis of data

Common dolphin bycatch was recorded mainly during fishing events targeting bigeye tuna (76% of all bycatch records) therefore we restricted the analysis to events with bigeye tuna. The software ArcGis 10.0 was used to produce geo-referenced maps to analyse spatial trends in common dolphin bycatch during the whole study period.

#### Operational and environmental variables

Generalized additive models (GAMs) were used to investigate the effect of fishing effort, fishing operations, tuna catches and environmental variables on the probability of dolphin bycatch and to determine which variables are the most adequate for stratification when applying ratio estimators (Table 1). GAMs were used to account for non-linear effects of predictors. GAMs predicting the presence/absence of bycatch used a binomial error distribution with an identity link function.

**Table 1:** Summary of explanatory variables for the GAM predicting the probability of dolphin bycatch.

Category	Explanatory variable	Type
Environmental	Sea surface temperature (°)	Continuous
	Depth (m)	Continuous
	Distance coast (km)	Continuous
	Prey abundance (t)	Continuous
Fishing operation	Hour of day	Continuous
	Gear	Categorical
	Latitude, longitude	Continuous
Fishing effort	Fishing duration (h)	Continuous
	Number of poles	Continuous
Catch	Number of tuna	Continuous
	Baitfish	Categorical
	Average tuna size (cm)	Continuous

The variables fishing duration, distance to coast, number of tuna caught, and average length of individual tuna were log transformed (+1 for covariates including zero values). Tuna weight (kg) and average weight of individual tuna (kg) were omitted from the models because they were strongly correlated.

Before fitting the models homogeneity, potential outliers and amount of zeros were analysed with Cleveland dotplots, boxplots, and frequency plots. Variance inflation factor (VIF) analysis and pairwise correlations were computed between all variables to check for collinearity (Zuur, Ieno & Elphick, 2010). A backward stepwise selection procedure was used to identify the best fitting model with the lowest Akaike’s Information Criterion (AIC) score. Model validation was applied on the best fitting model to verify the underlying assumptions (Zuur, Ieno & Smith, 2007). All calculations were conducted using R statistical package (R Core Team, 2015).

#### Bycatch Rates and Total Estimated Bycatch

We compared four different methods to estimate dolphin bycatch in the pole-and-line tuna fishery: (1) ratio estimate, (2) GAM describing dolphin bycatch rates, (3) negative binomial GAM, (4) generalized additive models for location, scale and shape (GAMLSS). Dolphin bycatch rates were calculated as the number of dolphins captured per observed tonnage of bigeye tuna landed per fishing trip. The fisheries official data report the landed weight of tuna by fishing trip and vessel but have no information on number of fishing events. Therefore, the only proxy of fishing effort available for the non-monitored section of the fleet was total bigeye tuna landed per trip (Silva et al., 2002, 2011). Dolphin bycatch rates calculated from monitored trips were then applied to total bigeye tuna landings by the whole fleet over the same time period to estimate the annual bycatch of dolphins in the pole-and-line bigeye tuna fishery.

##### (1) Ratio estimate method

Ratio estimation was used to extrapolate total bycatch from observed dolphin bycatch rates, using data stratified by sea surface temperature. SST was selected for stratification because it was a significant predictor of common dolphin bycatch (see below) and because information on SST was available in fisheries official data, enabling estimation of bycatch for the entire fleet. Based on the GAM that showed a decrease in bycatch probability at SST >18 °C, fishing data were grouped into two strata: high temperature (≥18 °C) and low temperature (<18 °C). A bycatch rate was calculated for each SST stratum as the sum of dolphins captured divided by observed tonnage of tuna landed. Stratum-specific bycatch rates were then multiplied by the total landings of the tuna fishery from 1998 to 2012 to calculate bycatch for the study period. Total bycatch over all strata correspond to the sum of the stratified estimates. Finally, the average annual bycatch is the total estimated bycatch over all strata divided by the number of years of the study (*n* = 15 yr). Confidence intervals (95% CI) of bycatch estimates were calculated using a non-parametric bootstrap procedure (Efron & Tibshirani, 1993) with fishing trip as a resampling unit.

##### (2) Generalized additive model

A GAM with a Poisson distribution was used to model the expected dolphin bycatch rate based on statistically significant explanatory variables. In order to use this model to calculate bycatch of the entire tuna-fishing fleet, only variables available in the POPA dataset and in the fisheries official data could be considered. Thus, explanatory variables included in the model were year and SST. Year was included in the model as a proxy for temporal variability to account for interannual differences in bycatch. Initial data exploration indicated that the observed counts were over-dispersed. Usually, models with a negative binomial (NB) distribution handle overdispersion better than Poisson regression models (Zuur, Ieno & Smith, 2007) so we decided to fit a second model with a NB distribution and a log link function to estimate the bycatch rate.

A backward stepwise selection procedure was used to identify the best fitting model using the AIC and variables with the highest AIC value were excluded. All continuous variables were considered as smooth terms in the model using the default degrees of freedom in the fitting procedure. Model validation was applied on the best fitting model to verify the underlying assumptions. Confidence intervals were calculated as for the ratio estimate method.

##### (3) Generalized additive models for location, scale and shape

The GAMLSS approach is a semi-parametric method that allows the relationship between the predictor variables and the response variable to be modelled either parametrically, with linear or nonlinear predictors, or non-parametrically, with smooth nonparametric terms (e.g. cubic splines or loess) and random effects (Rigby & Stasinopoulos, 2010). The GAMLSS can be considered as an extension of GAMs and generalized linear models. This method allows for a wide range of skewed and kurtotic distributions by explicitly modelling various distributional parameters, which may include the location/mean, scale/dispersion, skewness and kurtosis as functions of predictor variables (Rigby & Stasinopoulos, 2005).

Generalized additive models for location, scale and shape with a zero-adjusted gamma distribution (ZAGA) and zero-inflated beta distribution (BEZI) were used to model a continuous response and to deal with the excess of zeroes. These models were developed and implemented using gamlss package (Stasinopoulos & Rigby, 2007) in the R 3.1.2 software (R Core Team, 2015).

## Results

### Observed bycatch

From 1998 to 2012, 23,175 tuna fishing events were observed. Incidental captures occurred in 86 (∼0.4%) of these fishing events, with a total of 102 dolphins caught. Typically a single dolphin was captured per fishing event, with a maximum of three dolphins captured at the same time. Of the 102 dolphins, 92 were common dolphins, nine were Atlantic spotted dolphins (*Stenella frontalis*) and one bottlenose dolphin (*Tursiops truncatus*) (Table 2). Most (*n* = 78) of the common dolphin bycatch took place in bigeye tuna fishing events, mainly from 1998 to 1999 and in 2012. There were no reports of bycatch in 2003, 2004, 2007 and 2010 (Table 2). The majority of incidental catches took place in May and June. All dolphins were released alive (by cutting the fishing line) but the fate of these animals is unknown.

**Table 2:** Number of dolphins captured by the pole-and-line tuna fishery, monitored by POPA observers, from 1998 to 2012.

Year	Number of fishing events	Number of common dolphins	Number of Atlantic spotted dolphins	Number of bottlenose dolphins
1998	13	15	0	1
1999	23	18	7	0
2000	9	7	2	0
2001	1	1	0	0
2002	1	1	0	0
2003	0	0	0	0
2004	0	0	0	0
2005	5	6	0	0
2006	2	2	0	0
2007	0	0	0	0
2008	1	1	0	0
2009	2	2	0	0
2010	0	0	0	0
2011	7	9	0	0
2012	22	30	0	0
Total	86	92	9	1

**Figure 1:**
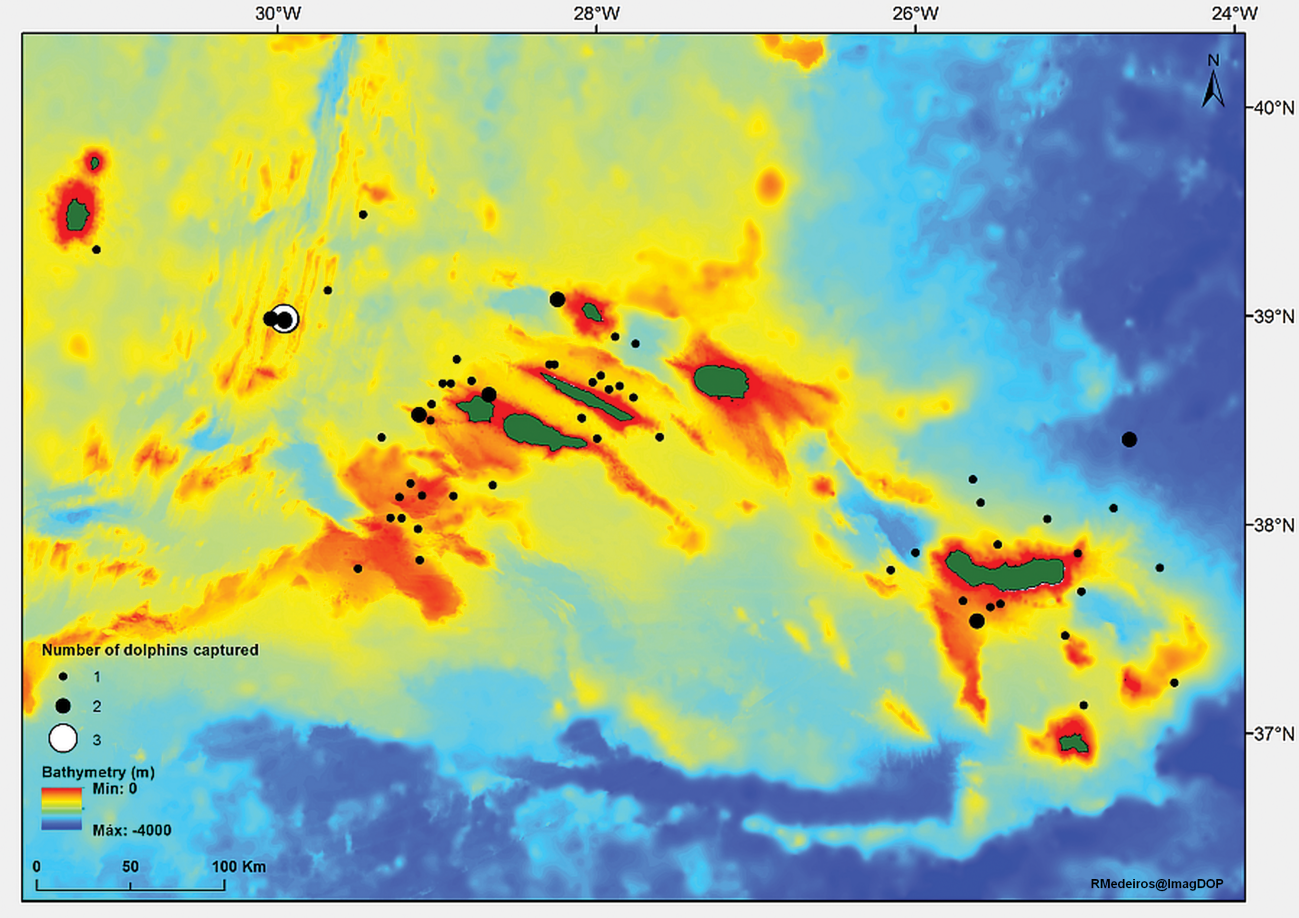
Location of bigeye tuna fishing events with common dolphin bycatch in the waters around the Archipelago of the Azores (in green) from 1998 to 2012.

Dolphin bycatch occurred throughout the archipelago but it was more frequent around the central and eastern islands (Fig. 1), where most of the fishing effort is concentrated (Cruz et al., 2016). A single dolphin was captured in the western group of islands.

### Predictors of common dolphin bycatch

The best fitting GAM showed that fishing duration, SST and geographic position (latitude and longitude) were significant predictors of bycatch probability (Table 3). Hour of day, gear, depth, distance to coast, number of fishing poles, prey abundance, number and average size of individual tuna did not influence bycatch probability.

**Table 3:** Summary of parameter estimates from the best-fitting GAM predicting probability of common dolphin bycatch in bigeye tuna fishery.

Explanatory variable	Non-parametric smoothers		
	edf	*X*^2^	*P*
s(Fishing duration)	1.604	59.10	<0.001
s(SST)	3.431	19.95	<0.001
s(long, lat)	5.818	18.13	0.020

**Note:**

edf, effective degrees of freedom.

**Figure 2:**
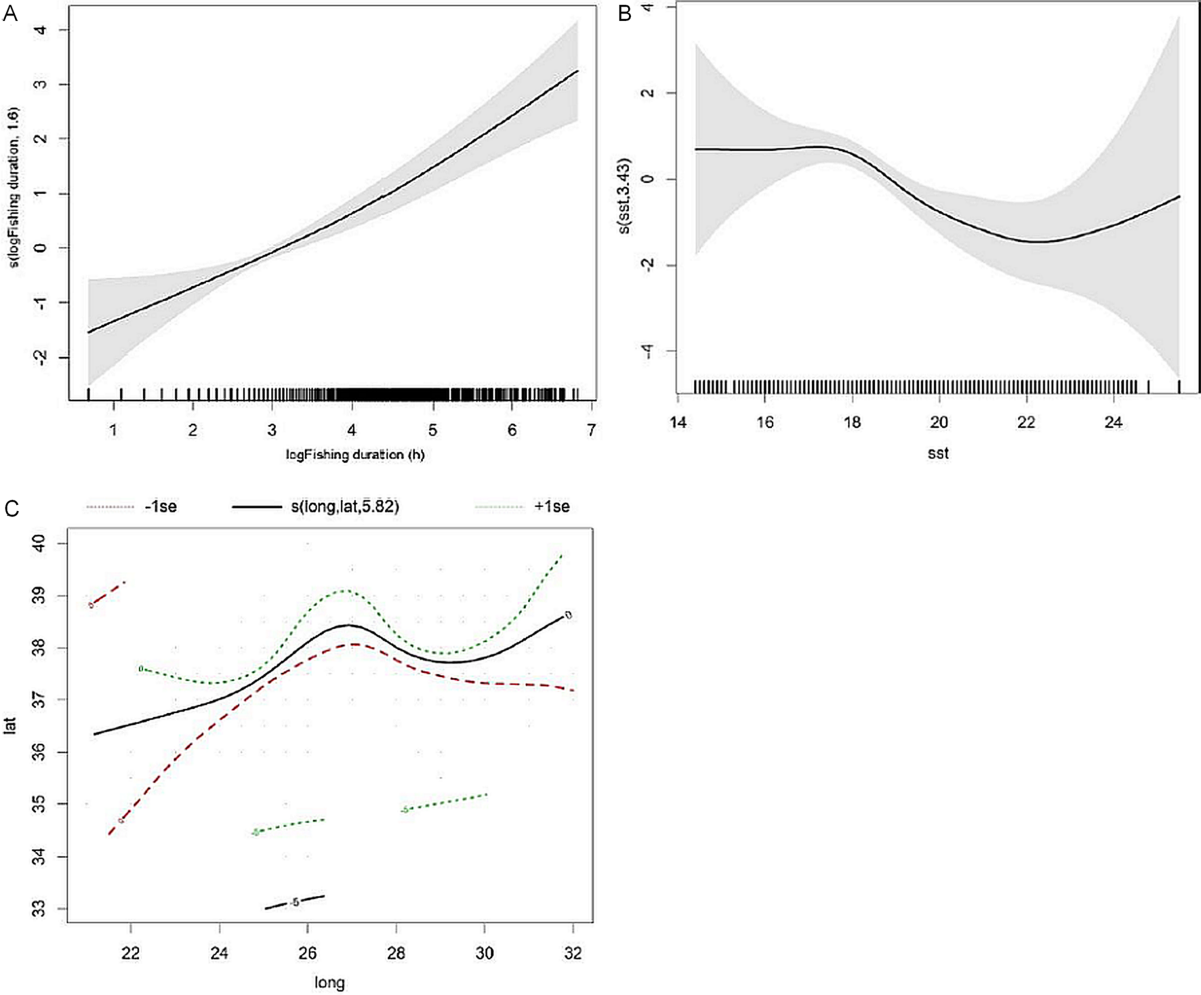
Smoothers estimates for the predictors (A) fishing duration, (B) SST and (C) spatial location (latitude/longitude) obtained by the GAM predicting probability of common dolphin bycatch. The grey shading indicates approximate 95% confidence bands. Tick marks on the x-axis are sampled data points.

The final GAM model explained 13.5% of the deviance. The model predicted that bycatch probability increased almost linearly with fishing duration and decreased in water temperatures higher than 18 °C. The likehood of dolphin bycatch increased towards the central group of islands (between 36° and 38° longitude) (Fig. 2).

### Estimation of total common dolphin bycatch

The GAM with a Poisson distribution and GAMLSS with a ZAGA and BEZI provided a poor fit to the data and gave inconsistent results. Therefore, we will only present results from the ratio estimate and NB GAM models.

#### Ratio estimate

This method estimated 73 (95% CI: 66–80) common dolphins captured at SST ≥18 °C and 123 (95% CI: 116–129) dolphins captured when SST was below 18 °C. Combining data from the two strata gives a total estimate of 196 (95% CI: 186–205) common dolphins captured by the tuna-fishing fleet between 1998 and 2012, and an average bycatch of 13 dolphins a year (Table 4).

**Table 4:** Ratio estimate of common dolphin bycatch for the bigeye tuna fishery from 1998 to 2012.

*T* (°C)	Observed bycatch	Observed catch (t)	Observed bycatch rate	Fleet catch (t)	Estimated bycatch	Average estimate per year
<18	26	1312.54	0.01981	3680.41	73	13 CV = 0.24 (95% CI: 7 to 9)
≥18	52	7469.24	0.00696	17622.73	123	

**Note:**

Bycatch rates are stratified by sea surface temperature.

#### Negative binomial GAM

**Figure 3:**
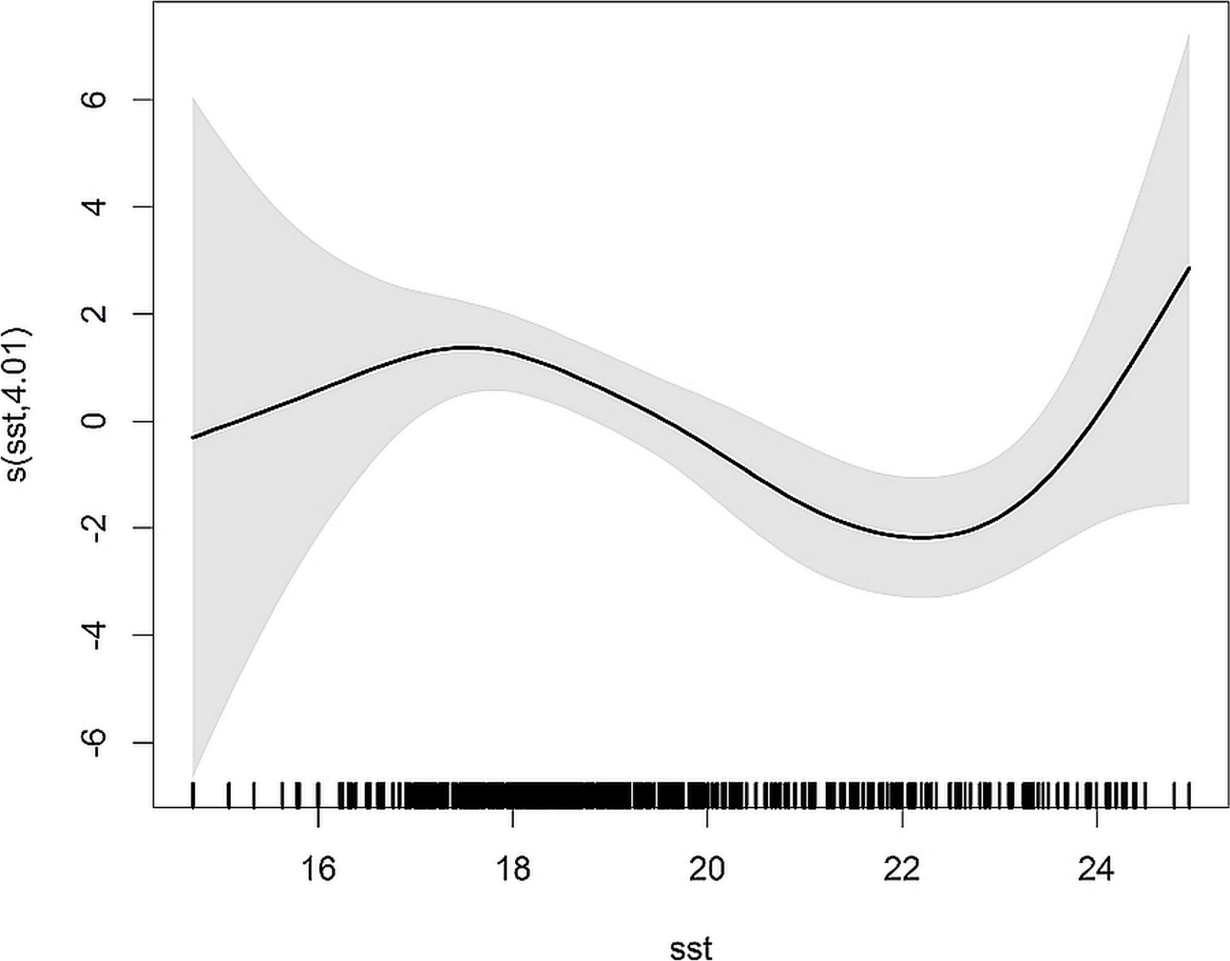
Smoothers estimate for the predictor SST obtained by the NB GAM predicting probability of common dolphin bycatch. The grey shading indicates approximate 95% confidence bands. Tick marks on the *x*-axis are sampled data points.

The model predicting dolphin bycatch with SST explained 12.8% of the deviance. Results from the negative binomial GAM agree well with the model predicting bycatch probability and indicated higher bycatch rates when SST was <18 °C (Fig. 3).

Estimated bycatch rates of common dolphins ranged between 0.004 and 0.022 per metric ton of bigeye tuna landed. Applying these bycatch rates to the entire the tuna fleet yielded an estimate of 262 (95% CI: 249–274) common dolphins bycaught from 1998 to 2012, and an average annual bycatch of 17 dolphins (CV = 0.23, 95% CI: 9–25). Estimates of annual bycatch ranged from 2 dolphins in 2003 to 50 animals in 2012 (Table S2).

**Figure 4:**
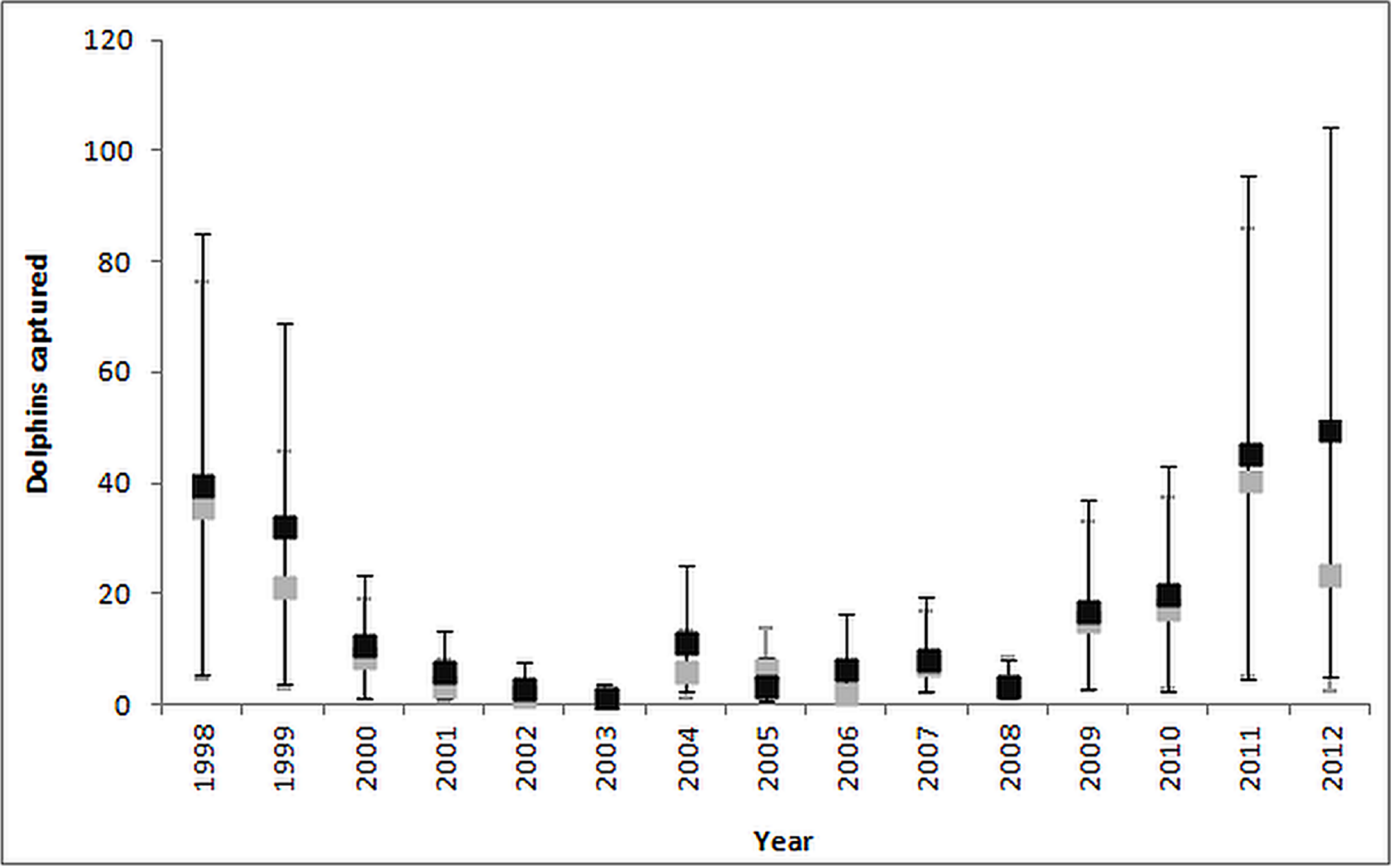
Comparison of ratio estimate (grey bars) and NB GAM (black bars) estimates of the annual common dolphin bycatch. The bars correspond to the 95% CI.

The estimated dolphin bycatch varied considerably by year for both the ratio estimate and negative binomial GAM approaches. Although these methods gave similar annual bycatch estimates, the NB GAM estimates were slightly higher particularly for the years of 2011 and 2012 (Fig. 4).

## Discussion

The analysis of a longer time series of fishery-observer data supports previous findings by Silva et al. (2002, 2011) that indicated low bycatch rates of small dolphins in the pole-and-line tuna fishery in the Azores. The large majority of cases of bycatch involved common dolphins. Common dolphins frequently interact with this fishery, by sinking the tuna schools and/or competing with tunas for live bait, namely bogue, sardine, chub mackerel and horse mackerel, in particular when the fishery targets bigeye tuna (Silva et al., 2002, 2011; Cruz et al., 2016).

The present study revealed that duration of fishing events, sea surface temperature and location (latitude and longitude) were significant predictors of common dolphin bycatch. The probability of dolphin bycatch increased with longer fishing events. Longer fishing events give dolphins increased opportunities for interacting with the fishery as reported by Cruz et al. (2016), thus increasing the likelihood that dolphins became hooked in the fishing line. The probability of common dolphin bycatch was higher at lower SST, which likely reflects the seasonal occurrence of common dolphins, and/or of bigeye tuna in the region. Common dolphins occur year-round in the Azores with higher encounter rates from winter to early summer (Silva et al., 2014). Similarly, catches of bigeye tuna in the Azores generally peak between May and July and decrease after this time (Dâmaso, 2007). While it is unclear if there is any relationship between the seasonality of the two species, their higher abundance in the area when SST is lower increases the likelihood of interactions (Cruz et al., 2016) and of dolphins being captured. The probability of dolphin bycatch was lower in the western islands and higher in the central and eastern islands. The combination of higher fishing effort and temporal occurrence of common dolphins in the latter regions may give rise to a bycatch hotspot. In spring and early summer, when common dolphins are more abundant in the Azores, the tuna fishing generally concentrates around the central and eastern group of islands and surrounding seamounts (Silva et al., 2002; Dâmaso, 2007; Cruz et al., 2016).

According to the ratio estimate, 196 common dolphins were incidentally captured by the tuna fleet in 15 years, while the results from the NB GAM gave 262 dolphins. The two methods gave similar annual bycatch estimates, though NB GAM-based estimates were higher especially in 2011 and 2012. Although we have no way of validating these estimates, the fact that two distinct analytical approaches provided similar annual results gives some confidence in their reliability and suggests that either of these methods may be used in the future to assess bycatch rates in this fishery.

However, these estimates may still be affected by a number of factors and there are a number of caveats that should be considered in this study. While the GAM indicated that location and duration of fishing events also influenced bycatch probability, these could not be used to estimate bycatch rates in the entire fleet because no information on these variables is available for the unobserved fleet. Given the substantial differences in bycatch rates between the three groups of islands, with the western islands showing almost no bycatch, incorporation of a variable describing fishing area could improve both the ratio and model-based bycatch estimates. Such information should be considered mandatory and incorporated into fisheries official data. Nonetheless, we do not expect this limitation to have significantly biased our estimates. First, because there is no reason to believe that the monitored and non-monitored components of the fleet behave differently with respect to fishing areas and operations, nor do we expect substantial differences in distribution of fishing effort between them. Second, because geographic differences in bycatch rates should, at least partly, reflect fishing effort, and this is accounted to some extent in our analyses.

A common critique of bycatch estimation methods is the use of landings as a proxy for fishing effort. Frequently there may be a poor relationship between catches of target species and bycatch of non-target species, since increasing bycatch may not necessarily be the result of increasing landings and therefore other units of effort should be considered. Also, changes in the availability of the target species over the study period may imply that more effort is needed to land the same amount of fish (Orphanides, 2009; Bjørge, Skern-Mauritzen & Rossman, 2013). In this study there was a positive relationship between bigeye landings and common dolphin bycatch, despite large interannual variations in landings. Although we acknowledge that duration of fishing events is more influential on dolphin bycatch than tuna catches and would be a more appropriate measure of fishing effort, this information was not available for vessels not monitored by observers and the only measure reported in the official data are landings per vessel and trip.

In the ratio estimate approach the paucity of observer data has been considered by some to bias bycatch rates due to constraints in observer coverage over time or across the fishing area (Lawrence et al., 2009). This should not be a major source of bias in our dataset, as POPA covers about 50% of the tuna-fishing vessels for the duration of the fishing season and all vessels use the same areas.

A number of approaches are available to model bycatch data that is over-dispersed relative to the Poisson distribution. In this study, the Negative binomial GAM performed better then the Poisson, GAMLSS ZAGA and BEZI distributions in terms of fit and predictive capabilities dealing with over-dispersed data and the large number of zeros.

Although estimates of common dolphin bycatch pertain only for the bigeye tuna fishery, observed bycatch in fishing events for other tuna species was very low (less than 20% of total observed bycatch). Therefore, we assume that number of common dolphins incidentally captured during events for other tunas do not significantly change estimates presented here. These bycatch estimates are very low compared with estimates from dolphin bycatch in fisheries elsewhere like in Peruvian and Ecuadorian artisanal fisheries (Mangel et al., 2010; Alava et al., 2017). In East Malaysia, where the fishing fleet is predominantly small-scale and coastal, bycatch was higher with about 527 dolphins taken annually (Jaaman, Lah-Anyi & Pierce, 2009).

In the Azores, the pole-and-line tuna fishery is the only fishery with records of dolphin bycatch. Silva et al. (2011) reviewed the interactions between cetaceans and fisheries in the Azores and reported no bycatch in demersal and swordfish fisheries. In the artisanal squid fishery there were also no observations or reports of incidental capture of Risso’s dolphins (Cruz et al., 2014). Yet monitoring of other fisheries, including the longline fishery, has been scarce. Marine mammal bycatch in the U.S. East Coast pelagic longline fishery is common, particularly of pilot whales (*Globicephala* spp.) and Risso’s dolphins and often results in the mortality or injury of these species (Garrison, 2007). Similarly, in the Hawaii and American Samoa longline fisheries, dolphins were seriously injured or died due to interactions with this fishery (Bradford & Foney, 2014). Thus, implementing an observation program to monitor the longline fishery in the Azores would be critical to assess bycatch patterns and drivers.

## Conclusion

Our study shows that bycatch of common dolphins in the Azorean pole-and-line tuna fishery is low and there is no evidence that bycatch rates have consistently increased over the 15-year period. Bycatch was primary driven by the spatiotemporal overlap between common dolphin and fishing effort. The higher bycatch rates occurred in specific geographic areas and at times favoured by dolphins and the fishery and bycatch probability increased with duration of fishing events, regardless of the time of day, fishing gear and local characteristics of the fishing site. As noted by Cruz et al. (2016), common dolphins and tunas have similar prey and likely exploit the same habitats. Live bait used by the tuna fishing vessels is an easy and readily available food source for common dolphins and dolphins are attracted to fishing vessels to prey on it. Under these circumstances, the most effective way to avoid bycatch may be for fishing vessels to stop fishing and move away from the area when dolphins are detected nearby. Other mitigation measures, such as use of acoustic deterrents, should be carefully considered, as the potential costs (addition of more noise into the marine environment, changes in tuna behaviour) may outweigh the benefits. Finally, future studies should consider complementing observer data with logbook data to refine or add new information about fishing operations in the estimation of bycatch.

## Supplemental Information

10.7717/peerj.4285/supp-1 Supplemental Information 1 Estimates of common dolphin bycatch for the bigeye tuna fishery from 1998–2012.

## Additional Information and Declarations

### Data Availability

The following information was supplied regarding data availability: Due to the sensitive nature of fisheries data and to confidentiality agreements with data providers, supporting data cannot be made openly available. Individual requests for the data can be sent to the POPA Coordination and Data Management (Miguel Machete; miguel.ag.machete@uac.pt) and will be addressed on a case-by-case basis.
