# Supplementary material for: Estimating common dolphin bycatch in the pole-and-line tuna fishery in the Azores
Source: PeerJ. 2018 Feb 12;6:e4285. doi: 10.7717/peerj.4285 (PMC5813588; doi:10.7717/peerj.4285)
Supplement: Supplemental Information 1 [file peerj-06-4285-s001.docx]

**Estimating common dolphin bycatch in the pole-and-line tuna fishery in the Azores**

Maria João Cruz^1,2^, Miguel Machete^2^, Gui Menezes^1,2^, Emer Rogan^3^ & Mónica A. Silva^2,4^

^1^Departamento de Oceanografia e Pescas, Universidade dos Açores, 9901-862 Horta, Portugal.

^2^MARE-Marine and Environmental Sciences Centre and IMAR – Instituto do Mar, 9901-862 Horta, Açores, Portugal.

^3^School of Biological, Earth and Environmental Sciences, University College Cork, Enterprise Centre, Distillery Fields, North Mall, Cork, Ireland

^4^Biology Department, Woods Hole Oceanographic Institution, Woods Hole, USA.

Corresponding Author:

Maria João Cruz

Email address: m.joao83@gmail.com

Supplementary table S1 – Estimated common dolphin bycatch for the bigeye tuna fishery from 1998-2012 with ratio estimate method.

| **Year** | **Estimated bycatch** |
| --- | --- |
| 1998 | 36 (31-40) |
| 1999 | 21 (19-24) |
| 2000 | 9 (8-10) |
| 2001 | 4 (3-5) |
| 2002 | 2 (1-2) |
| 2003 | 1 (1-2) |
| 2004 | 6 (5-7) |
| 2005 | 6 (5-7) |
| 2006 | 2 (2-3) |
| 2007 | 7 (5-9) |
| 2008 | 4 (2-5) |
| 2009 | 15 (13-18) |
| 2010 | 17 (14-20) |
| 2011 | 40 (35-46) |
| 2012 | 24 (21-26) |

Supplementary table S2 – Estimated common dolphin bycatch for the bigeye tuna fishery from 1998-2012 with NB GAM.

| **Year** | **Fleet catch(t)** | **Bycatch rate** | **Estimated bycatch** |
| --- | --- | --- | --- |
| 1998 | 3149.1 | 0.013 | 40 (34-45) |
| 1999 | 1507.5 | 0.022 | 33 (29-36) |
| 2000 | 784.9 | 0.014 | 11 (10-12) |
| 2001 | 349.6 | 0.017 | 6 (5-7) |
| 2002 | 225.2 | 0.015 | 3 (3-4) |
| 2003 | 206.3 | 0.007 | 2 (1-2) |
| 2004 | 876.4 | 0.013 | 11 (9-14) |
| 2005 | 912.4 | 0.004 | 4 (3-5) |
| 2006 | 329.4 | 0.021 | 7 (4-9) |
| 2007 | 1032.4 | 0.008 | 8 (6-11) |
| 2008 | 527.1 | 0.007 | 4 (2-5) |
| 2009 | 2189.6 | 0.008 | 17 (14-20) |
| 2010 | 1921.2 | 0.011 | 20 (18-23) |
| 2011 | 4189.2 | 0.011 | 46 (41-50) |
| 2012 | 3102.8 | 0.016 | 50 (45-55) |
